# Malaria rapid diagnostic tests for the case management of febrile children in Nigerian primary healthcare settings: a cross-sectional study

**DOI:** 10.1186/s42506-022-00105-5

**Published:** 2022-05-12

**Authors:** Phebe O. Ali, Joseph Okebe, Olabisi A. Oduwole

**Affiliations:** 1 Department of Medical Laboratory Science, Achievers University, Owo, Nigeria; 2 Institute of Tropical Diseases Research & Prevention, Calabar, Nigeria

**Keywords:** Children, Healthcare, HRP2, Malaria, Microscopy, mRDT, Plasmodium

## Abstract

**Background:**

Malaria has been identified as a significant public health burden, exhibiting a high risk of death and morbidity. In sub-Saharan Africa, most young children attending primary healthcare facilities are commonly diagnosed with malaria. Thus, introduction of malaria rapid diagnostic test (mRDT) kits and effective antimalarials has substantially improved the management of malaria cases. However, healthcare worker confidence and adherence to procedures dependent on malaria test results remain variable in high-burden settings due to lacking alternative point-of-care tests to diagnose other causes of fever. In this study, we compared the results of malaria screenings using mRDT and microscopy in febrile children presenting at a primary health facility.

**Methods:**

This study was conducted at a primary health center in Owo, Ondo State, Nigeria. Children with fever were assessed for malaria by health staff and, where indicated, screened using *Plasmodium falciparum* histidine-rich protein-2 mRDT kits. Blood samples were collected on slides for microscopy and in hematocrit tubes for hematocrit determination simultaneously, whereas the mRDT test was done by routine health staff. Children found positive for malaria via mRDT were diagnosed as uncomplicated malaria cases and treated as outpatients using artemether-lumefantrine. Blood slides were read independently by two trained microscopists blinded to the mRDT results. The parasite densities were defined as average counts by both microscopists. We then assessed the sensitivity, specificity, and predictive value of mRDT for the diagnosis of malaria.

**Results:**

We compared the test results of 250 febrile children who are under 15 years old. The test positivity rates were 93.6% (234/250) and 97.2% (243/250) using microscopy and rapid RDTs, respectively. The sensitivity and specificity of mRDT compared to microscopy were 100.0% and 43.8%, respectively, with a positive predictive value of 96.3% (95% CI 93.1–98.3). The hematocrit value was <30% in 64% of the children.

**Conclusion:**

As per our findings, mRDTs have correctly detected infections in febrile children. Healthcare workers and caregivers should be encouraged to act in accordance with the test results by means of regular feedback on the quality of mRDTs in use in malaria case management.

## Introduction

Malaria, known to be caused by *Plasmodium falciparum*, remains a significant public health challenge worldwide, with an estimated 229 million cases in 2019 in 87 countries where it is endemic [1]. These figures remain high despite the marked reductions in the global burden relative to estimates at the start of the millennium [2]. Between 2000 and 2019, it was estimated that 1.5 billion malaria cases and 7.6 million malaria deaths were averted, most of which were in sub-Saharan Africa [1, 3]. The World Health Organization (WHO) African Region accounts for 94% of cases and 95% of deaths globally [1]. Nigeria has the highest burden in this region, accounting for 27% of cases and 23% of deaths due to malaria [1]. Reductions in malaria burden were brought about by the introduction of policies and strategies, such as the use of long-lasting insecticidal bed nets (LLITN), improved malaria diagnosis, use of artemisinin-based combination therapy, and improved surveillance [4, 5].

The differential impact of these interventions contributed to changes in the epidemiological profiles of malaria risk and transmission [6, 7]. Some countries and regions have made significant reductions in coping with the disease burden and are considering elimination, while the burden of malaria has remained the same or is on increase in others.

The current protocols for malaria case management require screening for infection before treatment in cases with associated symptoms such as a history of fever [8]. Malaria rapid diagnostic test (mRDT) kits were introduced to improve confidence in malaria diagnosis; these have been shown to reduce over-diagnosis and over-prescription for malaria [9]. In areas where malaria transmission is stable, most children are infected in the first year of life, and severe disease is widespread in the first 2 years of life. In older children and adults, infections tend to remain asymptomatic due to the acquisition of a degree of clinical immunity [10, 11]. In high-burden areas, confidence in test results among healthcare workers remains a challenge due to the overlap of symptoms and signs between malaria and other causes of febrile illnesses and the high risk of re-infection [12]. To improve healthcare worker confidence in the use of malaria RDTs and adherence to national guidelines with reference to diagnosis-based treatment for malaria, local evidence with regard to the validity of these tests is needed and should be updated regularly.

Thus, in this study, we compared the utility of malaria RDTs in the diagnosis of malaria in children under 15 years old presenting with fever at a primary healthcare facility in an area with perennial and high malaria transmission intensity.

## Methods

### Study area and population

This cross-sectional study was conducted from August to October 2020 at the Oke-Mapo Primary Health Center in Owo, Ondo State, Southwestern Nigeria. The town is located 305 m above sea level and has a population of about 222,000. The clinic is generally focused on the treatment of common childhood infections with referrals for more serious conditions to the district and federal hospitals in Owo. Malaria treatment guidelines in Nigeria recommend screening individuals presenting with a history of fever in the past 48 h or having a measured temperature of >37.5°C for malaria using a *Plasmodium falciparum* histidine-rich protein 2 (HRP-2)-based mRDT kit or microscopy, followed by treatment with artemisinin-based combination therapy if the test returns positive [13]. The mRDT kit available at the clinic at the time of the study was the Standard Diagnostics (SD Bioline®), malaria Ag P.f RDT kit.

Informed consent were provided by the caregivers for children aged less than 15 years where the decision to screen for malaria was made by the attending healthcare worker. Once permission was given, we documented the child’s age, gender, and complaints on presentation. In addition to the blood sample collected for the mRDT test, we collected three additional drops of blood on a slide for later malaria screening via microscopy. The slides were labeled with the participant’s initials and screening number.

The blood smear was dried at room temperature and stained with 3% Giemsa stain for 30 min and was thereafter rinsed and dried. The stained slides were independently examined under the microscope by two laboratory scientists, and up to 100 high-power fields were viewed before the sample was declared negative. When a parasite was seen, quantification was done by counting parasites in relation to 200 white blood cells and assuming an average white cell count of 8000 cells/μL, following published protocols [14]. For the hematocrit (PCV) determination, microhematocrit tubes were filled to the predefined mark and centrifuged for 5 min at 11,000 rpm in a microhematocrit rotor (Hawksley and Sons Ltd, Sussex, UK), and the PCV value was measured with a hematocrit reader.

To determine the sample size for this study, we assumed a 5% discordance in results between microscopy [15] and mRDT. We estimated that in 250 paired samples, we would be able to detect this level of discordance with a 90% power and a 5% significance level. This number included an additional 10% increase in the estimate to account for samples that were destroyed during processing.

Data collected on paper forms were entered in an access database and were verified against the source document. We presented a description of the study population and the association between malaria covariates, including age and gender, assessed using a regression model. We also determined the sensitivity, specificity, and predictive values of mRDT compared to microscopy.

## Results

**Fig. 1:**
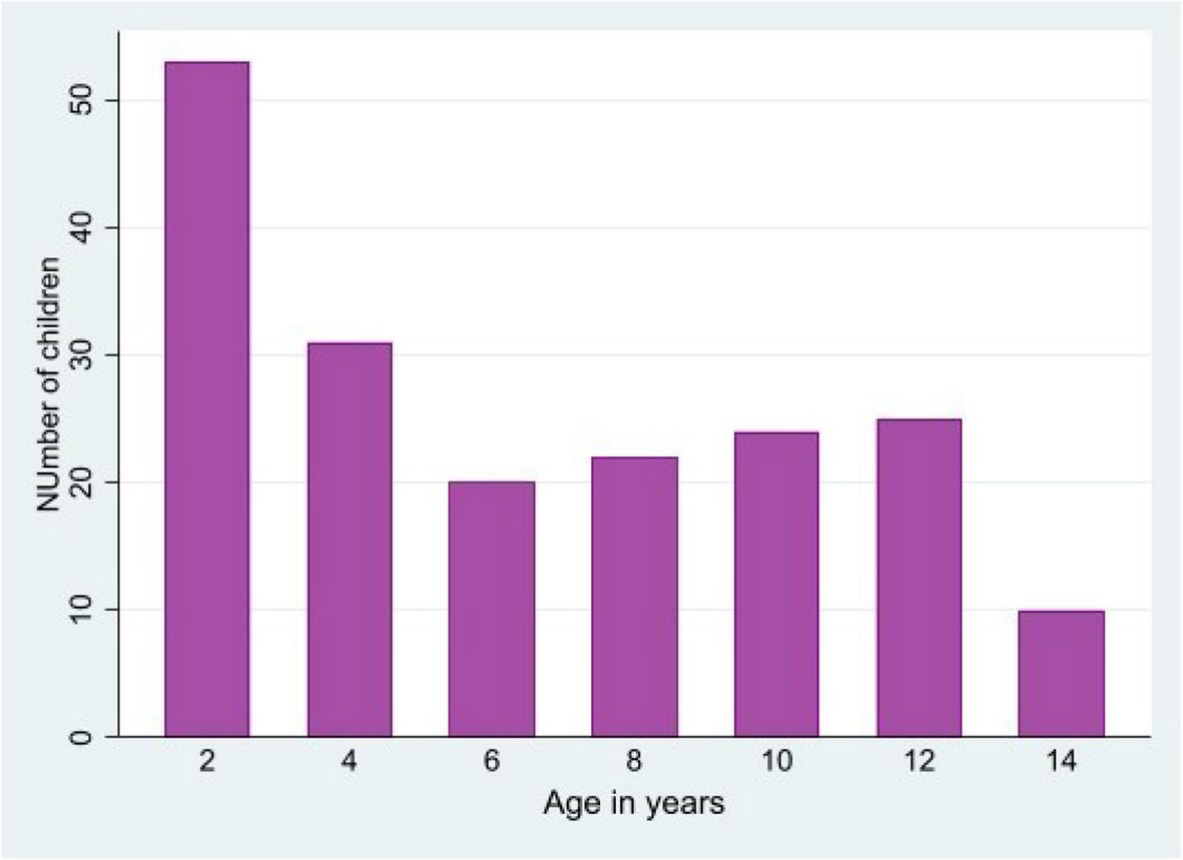
Age distribution of febrile children attending a primary health center in Owo, Nigeria (*n*=250)

We screened 250 children with a history of fever for malaria using both mRDTs and microscopy. Of those enrolled, 46.0% (115/250) were female (Table 1). The mean (standard deviation, [SD]) age of the children was 5.52 years (±4.34), with 53.6% (134/250) aged less than 5 years (Fig. 1). The mean (SD) packed cell volume in the cohort was 28.9% (±5.8); there was no significant difference in the mean PCV in children aged below and above 5 years (mean 29.1% (±5.7) and mean 28.7% (±5.8) were recorded respectively; *P*=0.84), with 63.4% (159/250) of children having PCV < 30%.

**Table 1:** Baseline characteristics of febrile children attending a primary health center in Owo, Nigeria, and malaria test results by mRDT and microscopy

	Under 5 years	Over 5 years	Total
Number	134 (53.6%)	116 (46.4%)	250 (100.0%)
Mean age (SD) in years	1.90 (1.17)	9.41 (2.95)	5.52 (4.34)
Female	63 (47.0%)	52 (44.8%)	115 (46.0%)
Mean (SD) packed cell volume (%)	29.1 (5.7)	28.7 (5.8)	28.9 (5.8)
RDT positive	129 (96.3%)	114 (98.3%)	243 (97.2%)
Microscopy positive	123 (91.8%)	111 (95.7%)	234 (93.6%)
Mean (SD) parasite density in positive tests, /μL	2365 (2682)	2536 (2588)	2446 (2634)

From the mRDT results, 97.2% (243/250) were found to be positive for malaria and were treated with artemether-lumefantrine; 53.1% (129/243) were children younger than 5 years. Children over 5 years were twice more likely to have had a positive test (OR 2.21, 95% CI 0.42–11.61, *p* = 0.349) than those younger than 5 years.

Microscopy screening showed that 93.6% (234/250) were positive, with a mean (SD) parasite density of 2446 (2634))/μL. Children over 5 years old were more likely to have a positive slide result (OR 1.99, 95% CI 0.67–5.89, *p* = 0.217), although mean parasite densities were not different between these age groups after adjusting for sex and PCV level (adjusted OR 1.1, 95% CI 0.96–1.26, *p* = 0.166).

A comparison of test results between mRDT and microscopy showed them to be consistent in 241 paired samples: 234 positive and 7 negative (Spearman’s Rho 0.649, *p* < 0.001). The sensitivity and specificity of mRDT relative to microscopy were 100.0% and 43.8%, respectively (Table 2). The positive and negative predictive values for mRDTs were 96.3% (95% CI 93.1–98.3) and 100.0%, respectively.

**Table 2:** Comparison of malaria test results for RDT and microscopy

	Microscopy		Total
	Positive	Negative	
RDTs			
Positive	234 (96.3%)	9 (3.7%)	243
Negative	0	7 (100.0%)	7
Total	234 (93.6%)	16 (6.4%)	250

## Discussion

Malaria diagnosis is available in most primary healthcare settings because of the ease of use and the stability of RDT cassettes, across a range of temperature and humidity. Results are also easy to interpret. In 2012, the WHO launched the *Test, Treat, Track (T3) initiative to* scale up diagnostic testing, treatment, and surveillance for malaria in endemic countries. This program was developed to ensure that all malaria patients receive prompt treatment. Furthermore, T3 was developed to improve surveillance for malaria cases and deaths and help Ministries of Health to determine which areas or population groups are most affected and help target resources where they are most needed. However, the use of malaria diagnostics remains to be limited, and opinions are divided on its utility [16, 17].

In this study, children presenting with fever in the past 48 h or having a measured temperature of >37.5°C were screened using routine *Pf*HPR2 mRDT and microscopy. Children with positive mRDT results were diagnosed as having uncomplicated malaria cases and were treated by routine healthcare workers with artemether-lumefantrine and discharged as outpatients following the national treatment guideline. The test positivity rate for malaria using microscopy was 93.6%, with slightly higher rates in children over 5 years old (95.7%. 111/116). Older children also had higher mean parasite densities than those who were under 5 years old. The sensitivity of the mRDT was 100%, with a positive predictive value of 96.3%.

Furthermore, approximately 64% of the children examined were moderately anemic with a hematocrit <30% [18]. Malaria is an important cause of anemia in endemic areas, as seen from the hemolysis of infected and uninfected red blood cells [19]. In high malaria transmission settings such as Nigeria, most young children and many older children and adults’ exhibit reduced hemoglobin concentration due to malaria infection [20]. The finding of this research underscores the need to screen children with fever presenting at primary healthcare for malaria as well as anemia in malaria-endemic areas.

Jegede et al. showed that an integrated intervention involving RDTs and Artemisinin-based combination therapy (ACTs) by community health workers (CHWs) in Burkina Faso, Nigeria, and Uganda was feasible and acceptable to their communities [21]; however, there are concerns about providing treatment to children with a false-positive RDT test, resulting in the over-prescription of antimalarials and antibiotics [22, 23]. However, it has been found that withholding antimalarials from children with a negative test is safe even in highly endemic areas [18]. The patterns of malaria transmission continue to evolve as the awareness and use of control measures increase. Studies such as this one are useful because healthcare workers and caregivers need to be aware of the relative risk of malaria infection in their settings to sustain their confidence in the results of malaria tests and reduce the rates of misdiagnosis and over-prescription of antimalarials and antibiotics.

### Study limitations

This study did not investigate other causes of fever in the participating children and there was no follow-up of treated participants to determine outcome after treatment.

## Conclusion

Malaria remains to be a significant cause of clinic presentations for febrile illness in children under 15 years old. Malaria RDTs are very sensitive and are determined to correctly predict infection in children. Healthcare workers and caregivers should be encouraged to adhere to test results in the case management of malaria in highly endemic settings.

## Data Availability

Data set used will be made available on reasonable request to the corresponding author.

## Abbreviations

CHWs: Community health workers
mRDT: Malaria rapid diagnostic test
WHO: World Health Organization
HRP-2: Histidine-rich protein 2
PCV: Packed cell volume
Pf: *Plasmodium falciparum*
